# Prediction of Postoperative Survival in Young Colorectal Cancer Patients: A Cohort Study Based on the SEER Database

**DOI:** 10.1155/2022/2736676

**Published:** 2022-07-04

**Authors:** Sheng Pan, Wenchao Mei, Linfei Huang, Yan'e Tao, Jing Xu, Yuelu Ruan

**Affiliations:** Puren Hospital Affiliated to Wuhan University of Science and Technology, Wuhan, 430000 Hubei, China

## Abstract

**Objective:**

Our aim is to make accurate and robust predictions of the risk of postoperative death in young colorectal cancer patients (18-44 years old) by combining tumor characteristics with medical and demographic information about the patient.

**Materials and Methods:**

We used the SEER database to retrieve young patients diagnosed with colorectal cancer who had undergone surgery between 2010 and 2015 as the study cohort. After excluding cases with missing information, the study cohort was divided in a 7 : 3 ratio into a training dataset and a validation dataset. To assess the predictive ability of each predictor on the prognosis of colorectal cancer patients, we used two steps of Cox univariate analysis and Cox stepwise regression to screen variables, and the screened variables were included in a multifactorial Cox proportional risk regression model for modeling. The performance of the model was tested using calibration curves, decision curves, and area under the curve (AUC) for receiver operating characteristic (ROC).

**Results:**

After excluding cases with missing information (*n* = 23,606), a total of 11,803 patients were included in the study with a median follow-up time of 45 months (1-119). In the training set, we determined that ethnicity, marital status, insurance status, median annual household income, degree of tumor differentiation, type of pathology, degree of infiltration, and tumor location had independent effects on prognosis. In the training dataset, taking 1 year, 3 years, and 5 years as the time nodes, the areas under the working characteristic curve of subjects are 0.825, 0.851, and 0.839, respectively, and in the validation dataset, they are 0.834, 0.837, and 0.829, respectively.

**Conclusion:**

We trained and validated a model using a large multicenter cohort of young colorectal cancer patients with stable and excellent performance in both training and validation datasets.

## 1. Introduction

Patients with colorectal cancer are at significant risk of death after surgery [1]. Postoperative mortality is widely used as a measure of professionalism and safety of hospitals, clinical teams, and surgeons [2]. Such studies addressing quality of care are increasingly being conducted with the aim of promoting quality of care improvement, identifying optimal treatment decisions, and helping patients improve their prognosis [3, 4]. These processes need to be adjusted for differences in patient mortality risk, and an accurate and reliable method of stratifying patients for mortality risk is needed to ensure that high-risk patients receive appropriate care and stabilize the physician-patient relationship [5].

The World Health Organization's Global Cancer Observatory (GLOBOCAN) counted more than 1.9 million new cases of colorectal cancer (including anal) and nearly 935,000 deaths in 2020. Incidence rates in younger age groups (age at diagnosis < 50 years) are increasing by 1-4% per year [6]. The US Preventive Services Task Force (USPSTF) suggests that because current information on risk factors is based almost exclusively on data from older adults, further research is needed to elucidate the underlying causes of colorectal cancer development for younger age groups [7]. This may be because older adults are overrepresented in colorectal cancer patients. The vast majority of studies now exploring the prognosis of colorectal cancer patients also do not strictly limit age [8–11], and even less literature has explored the postoperative survival rates affecting younger colorectal cancer patients, which is an issue well worth exploring. Our study limited the age of the study cohort to 18-44 years (defined by WHO as young adults), which makes our study more relevant. Older colorectal cancer patients are characterized by physical deterioration, more concomitant diseases, and slower postoperative recovery period compared with younger colorectal cancer patients [12]. Limiting age can also reduce the impact of interfering factors accompanying aging to a certain extent. Also, since younger colon cancer patients have a longer expected survival period, early intervention would be more meaningful and have better outcomes.

The data available to us is the Surveillance, Epidemiology, and End Results Program (SEER) (https://pubmed.ncbi.nlm.nih.gov/?term=SEER), a large database where the registry regularly collects follow-up information on patient demographics, tumor characteristics, and vital status, covering 30.0% of African Americans, 44% of Hispanics American, 49% of American Indians and Alaska Natives, 57.5% of Asians, and 68.5% of Hawaiian/Pacific Islanders. African Americans, 44% Hispanics, 49.3% American Indians and Alaska Natives, 57.5% Asians, and 68.5% Hawaiian/Pacific Islanders (https://pubmed.ncbi.nlm.nih.gov/?term=SEER). The multicenter nature of the data and the large sample size increase the generalizability of the model. A related method that can also be utilized is the nomograph, which, unlike complex machine learning models, can better stratify risk and possess simplicity and ease of interpretation.

## 2. Method

### 2.1. Study Cohort

We used the SEER database to retrieve patients diagnosed with colorectal cancer between 2010 and 2015 as the study cohort. The inclusion criteria for patients were as follows: (1) histologically confirmed colorectal cancer, classified according to the International Classification of Diseases for Oncology (ICD-O-3); (2) age between 18 and 44 years old (the World Health Organization defines 18-44 years old as young); and (3) close follow-up and survival information available; (iv) having undergone colorectal surgery. For each patient, we extracted information including (1) patient information: ethnicity, gender, survival time, survival status, insurance/marital status, and median household income (2011-2015); (2) tumor information: tumor location, degree of differentiation, histological pattern, invasive status, total number of malignant tumors, total number of benign/junctional tumors, and whether it was first in situ cancer; and (3) treatment information: surgery status, radiotherapy status, and cause of death.

### 2.2. Feature Selection

To assess the predictive power of each feature, we screened all features in two steps (*P* < 0.05 was considered statistically significant).

**Step 1 .**

The correlation of variables with patient prognosis was explored by means of Cox univariate analysis (using the ezcox R software package: version 1.0.2), and features that were not statistically significant were removed.

**Step 2 .**

To streamline the model, the variables retained from Step 1 were included in the Cox stepwise regression (independent variables were entered using the forward: LR method) for another screening.

### 2.3. Predictions and Verifications

The Cox regression model was trained using the survival R package (version 3.2.13) to predict the risk of death in young colorectal cancer patients 1, 3, and 5 years after surgery. After the model was built, cases from the training and validation sets were included in the model for validation. The predictive values of the model were calculated, and ROC curves, clinical decision curves, and calibration curves were plotted to check the efficacy of the model. All statistical analysis processes are carried out in the R 4.1.2 Programming language (https://www.r-project.org/).

## 3. Result

### 3.1. Data Description

We excluded cases with missing information (*n* = 23,606) and ended up with 11,803 patients included in the final study cohort with a median follow-up time of 45 months (1-119). The cohort of patients included in the study was randomly partitioned into training and validation datasets in a 7 : 3 ratio. There were 8297 patients in the training dataset (4240 men and 4057 women) and 3506 patients in the validation dataset (1832 men and 1674 women), with a median survival time of 45 months (1-119) for both datasets. (Table 1).

### 3.2. Feature Selection

The results of the Cox univariate analysis showed that the variables of age, insurance status, marital status, median annual household income, race, tumor location, degree of differentiation, type of pathology, and depth of infiltration had an independent effect on the prognosis of young colorectal cancer patients after surgery. The variables that were statistically significant in the univariate analysis were included in the Cox stepwise regression, and the analysis showed that insurance status, marital status, median annual household income, race, tumor location, degree of differentiation, type of pathology, and depth of infiltration had stronger independent effects on the prognosis of young colorectal cancer patients after surgery and were included in the model as characteristics of this study. (Table 2).

### 3.3. The Establishment of the Nomogram

**Figure 1:**
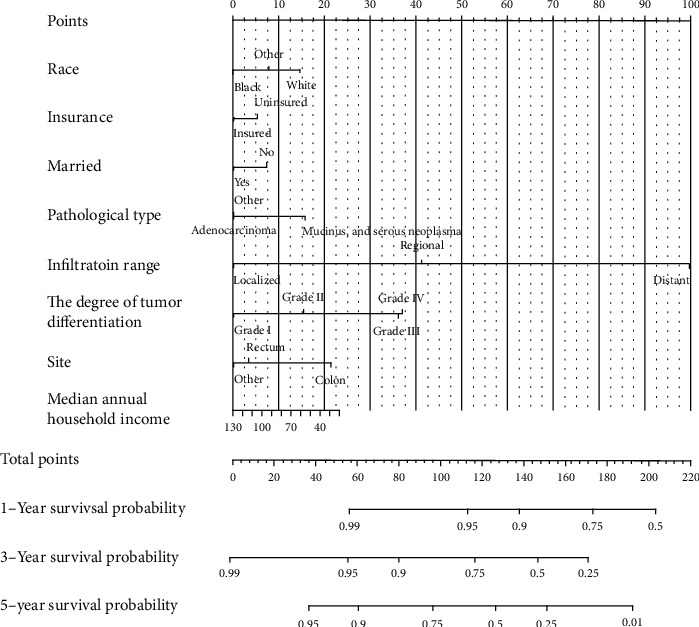
The nomogram of the Cox regression model.

The screened features were included in the Cox regression model for modeling (Table 3), and nomographs were drawn (Figure 1). the *C*-index of the Cox model was 0.812.

### 3.4. Models' Performances

**Figure 2:**
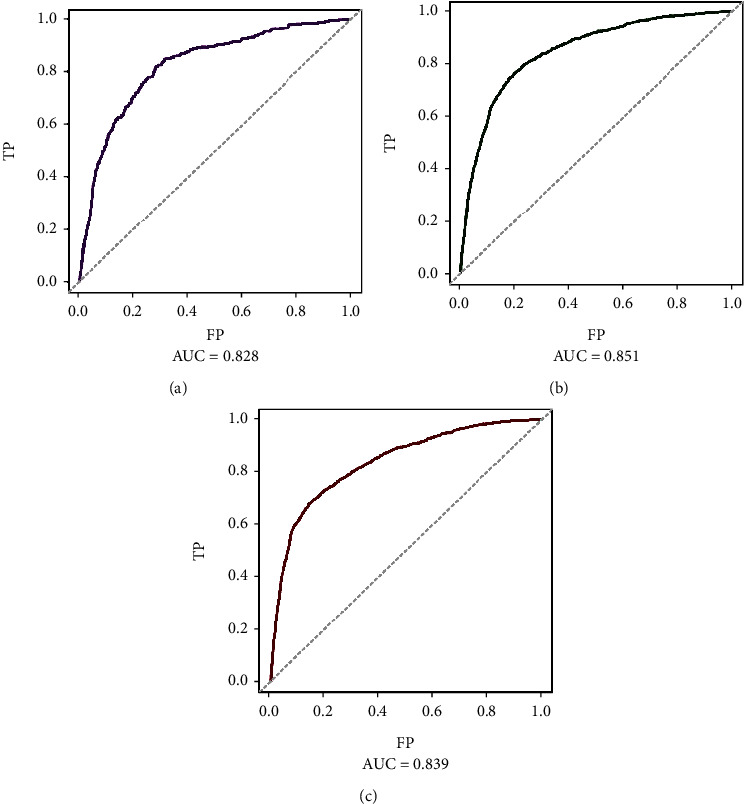
The ROC curves of the training dataset: (a) the mortality within one year; (b) the mortality within three years; (c) mortality within five years.

**Figure 3:**
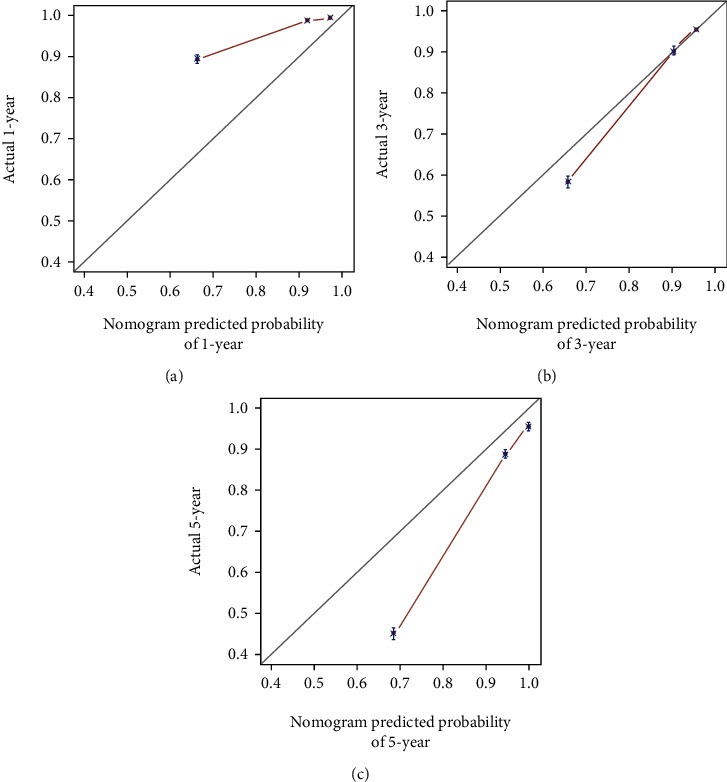
The calibration curves in the training dataset (from left to right are the calibration curves for the prediction of survival in year 1, year 3, and year 5, respectively).

**Figure 4:**
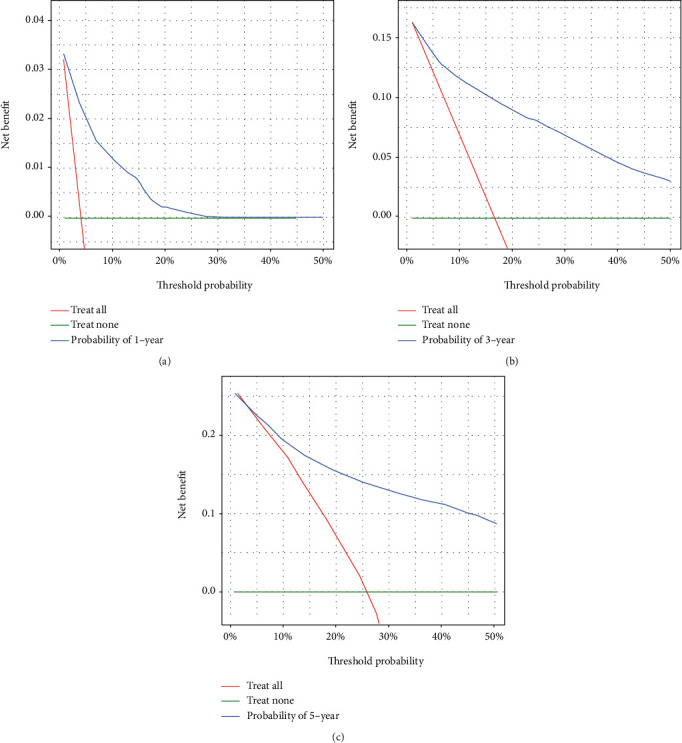
The decision curves in the training dataset (from left to right are the decision curves for the prediction of survival in year 1, year 3, and year 5, respectively).

In the training dataset, the areas under the ROC curves for the predicted values of 1-year, 3-year, and 5-year survival were 0.825, 0.851, and 0.839, respectively (Figure 2). The calibration curves (Figure 3) and clinical decision curves (Figure 4) are as follows.

**Figure 5:**
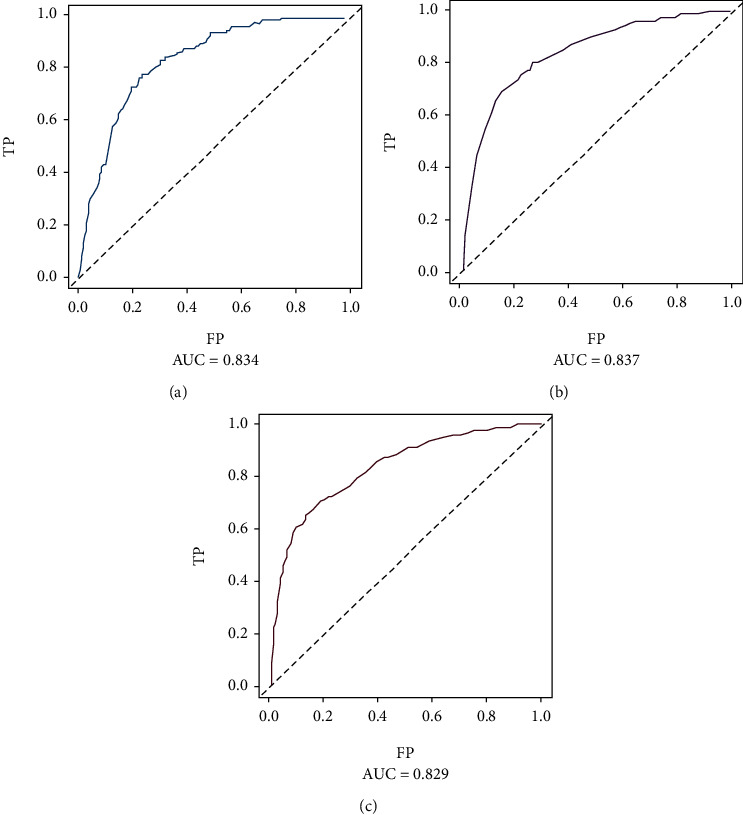
The ROC curves of validation dataset: (a) the mortality within one year; (b) the mortality within three years; (c) mortality within five years.

**Figure 6:**
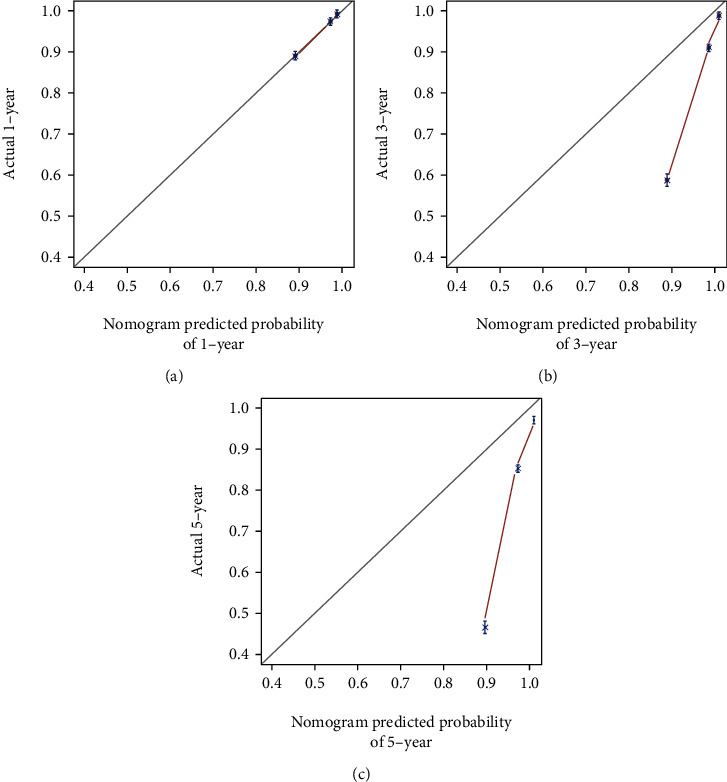
The calibration curves in the validation dataset (from left to right are the calibration curves for the prediction of survival in year 1, year 3, and year 5, respectively).

**Figure 7:**
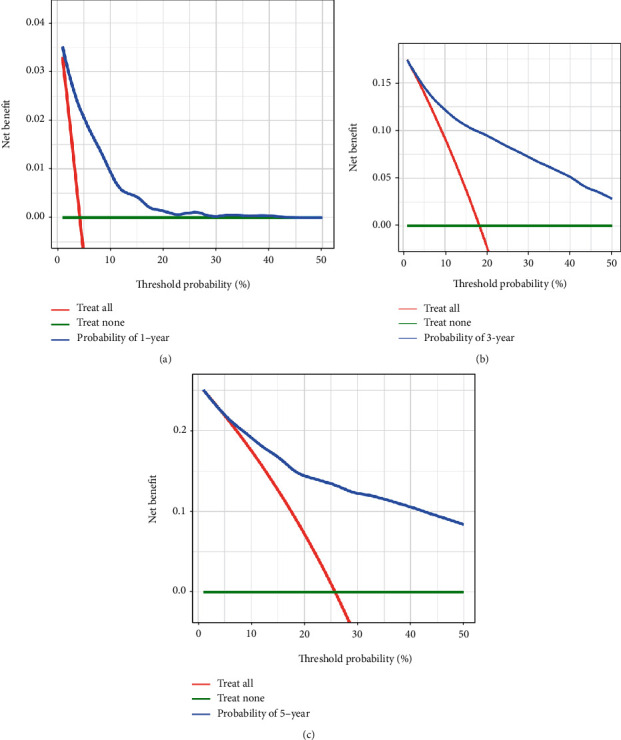
The decision curves in the validation dataset (from left to right are the decision curves for the prediction of survival in year 1, year 3, and year 5, respectively).

In the validation dataset, the areas under the ROC curves for the predicted values of 1-year, 3-year, and 5-year survival were 0.834, 0.837, and 0.829, respectively (Figure 5). The calibration curves (Figure 6) and clinical decision curves (Figure 7) are as follows.

## 4. Discussion

In this study, we screened young colorectal cancer patients aged 18-44 years from the SEER database; developed a Cox regression model based on patient demographic information, radiotherapy status, and tumor characteristics; and plotted a nomograph. It was used to assess the prognostic relevance of each factor to predict the prognosis of young colorectal cancer patients. The results provided reliable evidence of the predictive power of key risk factors, showing that insurance status, marital status, median annual household income, race, tumor location, degree of differentiation, type of pathology, and depth of infiltration were all statistically significant predictors of prognosis in young colorectal cancer. The nomograph model built with these predictors had excellent predictive effect, and the area under the ROC curve for the predicted values of 1-year, 3-year, and 5-year survival was 0.825, 0.851, and 0.839 in the training dataset, respectively. In the validation dataset, the areas under the ROC curves for the predicted values of 1-year, 3-year, and 5-year survival rates were 0.834, 0.837, and 0.829, respectively.

In terms of study cohorts, current information on risk factors is based almost exclusively on data from older adults [7], whereas our study limited the age of the patient cohort to 18-44 years, which to some extent fills the gap in existing research data. A study has shown that the proportion of short-term postoperative complications did not differ significantly between the older patients and younger patients (*P* = 0.097) [13]; however, due to the different bodily functions of elderly patients and young patients, the development of the long-term physical condition in the two types of patients will be different. Both overall survival and disease-specific survival (DSS) rates declined with advancing age; this may be due to elderly patients' poor physical recovery and their chronic disease [14]. Additionally, beneficial clinical decision making is particularly important for younger patients, as favorable treatment plans tend to have more pronounced effects due to the better physical fitness of young patients.

In terms of predictors, our study has two advantages in the selection of predictors: first, the predictors incorporated in our training model are easily available. Medical and demographic information is recorded during the patient's hospitalization, while information on tumor characteristics (degree of differentiation, pathological type, and depth of infiltration) is also recorded after surgery. This suggests that our model will not have much difficulty in generalization and has relatively wide applicability. Second, the predictors selected for our study are stable in nature. Several studies have shown that host-driven inflammatory responses contribute significantly to tumor behavior and treatment outcome [15, 16]. Tumor growth and metastatic spread are the result of interactions between tumor and mesenchymal factors, including blood vessels, inflammatory cells, and the immune system [17, 18]. Laboratory markers that lead to systemic inflammatory responses, such as CRP, hypoalbuminemia, white blood cell count, neutrophil/lymphocyte ratio (NLR), or platelet/lymphocyte ratio, have been shown to be prognostic and predictive factors for several tumors [19, 20]. However, inflammatory markers vary considerably between individuals with the same disease course or between different disease courses in the same individual. The inclusion of unstable laboratory markers in a predictive tool is likely to have a negative impact and, instead of contributing to prediction, may become a confounding factor for prediction. The variables included in this study (tumor characteristics, medical treatment, and demographic information) were relatively stable and adapted to different individuals with different disease courses.

In addition to this, we included social factors such as insurance status, marital status, and median annual household income, and the results showed that uninsured, single/divorced marital status, and low household income increased the risk of death among patients, which was consistent with our expected results. Marital status responds to some extent to the psychological status of the patient, while other social factors respond to the economic and social security status of the patient. Many studies have shown that mortality and morbidity for each disease are related to the economic status of the patient [21, 22] because economic status tends to reflect the quality of medical care received by the patient, the level of medical technology developed in the patient's region, and the cost of medical care for the patient [23].

In terms of research methodology, our study used nomographs drawn based on Cox regression as a prediction tool rather than machine learning models. Artificial neural networks, random forests, and support vector machine models, which are widely used with the advantage of fitting nonlinear relationships [24–26], may sound superior to models that can plot nomographs (Cox regression, logistic regression). However, the application of most machine learning models is limited to the research itself, and there are several obstacles in their path to widespread use: first, the “black box” effect of machine learning models is difficult to explain and to gain the trust of clinicians. Second, most of the studies did not result in a user-oriented application interface, only a narrative of their process and results. In addition, some researchers published the code and data of their models to a public website, but these codes were difficult to reproduce due to differences in programming environments or incomplete codes. In summary, with good performance, it is optimal to apply nomographs as a prediction tool, which allows clear hazard stratification. Most importantly, it has the advantage of visualization, which greatly increases the interpretability of the model and facilitates its generalization and application.

Our model has an additional advantage over other models. Some published models may be overly optimistic in their estimates of model efficacy. They often show excellent performance in the training dataset, but their discriminative power in the validation dataset is usually much lower than that in the training dataset [27–30]. Other studies have shown significant overfitting due to the small sample size of the data [31, 32], while our model has limited bias due to overfitting due to sufficient amount of data. From the results, it seems that its performance is excellent and robust in both the training and validation datasets.

There are some limitations to this study: first, our study cohort was based on the SEER database, which was not designed for our experimental purposes and had limited predictors to include, missing some of our variables of interest. However, there are some advantages of using the SEER database. First, the SEER database has a large sample size and can provide a sufficiently large dataset for the study. Second, SEER, as a public database, has a high level of confidence in the data. In addition, our model has only used retrospective data as a validation dataset and has not been prospectively validated, which requires a longer period of close follow-up and is the next step in our study.

## 5. Conclusion

We developed a simple, interpretable nomograph model that can accurately predict the prognostic status of young colorectal cancer patients after surgery, with robust clinical performance. The findings showed that, by tumor location, the risk of death was greater for colon cancer than rectal cancer and greater for cystic/mucinous colon cancer than for colorectal adenocarcinoma, and undifferentiated (Coef = 1.0637, *P* < 0.0001) and poorly differentiated cancer (Coef = 1.0353, *P* < 0.0001) would increase the risk of death in patients with colorectal cancer, and the degree of risk was comparable.

## Figures and Tables

**Table 1:** Data description.

	Validation set (*n* = 3506)	Training set (*n* = 8297)	*P*
Status = dead (%)	790 (22.5)	1844 (22.2)	0.7321
Survival time (mean (SD))	51.51 (32.07)	51.03 (31.89)	0.4582
Age (mean (SD))	21.21 (5.35)	21.15 (5.43)	0.5841
Race (%)			0.4173
White	2626 (74.9)	6309 (76.0)	
Black	484 (13.8)	1097 (13.2)	
Other	396 (11.3)	891 (10.7)	
Sex = female (%)	1674 (47.7)	4057 (48.9)	0.2624
Insurance = yes (%)	2165 (61.8)	5159 (62.2)	0.6770
Site (%)			0.6911
Colon	2150 (61.3)	5039 (60.7)	
Rectum	1255 (35.8)	3032 (36.5)	
Other^a^	101 (2.9)	226 (2.7)	
The degree of tumor differentiation (%)			0.4660
Grade I	363 (10.4)	811 (9.8)	
Grade II	2415 (68.9)	5691 (68.6)	
Grade III	613 (17.5)	1537 (18.5)	
Grade IV	115 (3.3)	258 (3.1)	
Pathological type (%)			0.3432
Adenocarcinomas	3073 (87.6)	7268 (87.6)	
Cystic, mucinous, and serous neoplasms	316 (9.0)	788 (9.5)	
Other^ab^	117 (3.3)	241 (2.9)	
Infiltration range (%)			0.2313
Localized	1206 (34.4)	2737 (33.0)	
Regional	1638 (46.7)	3906 (47.1)	
Distant	662 (18.9)	1654 (19.9)	
Lymph node dissection = yes (%)	2455 (70.0)	5820 (70.1)	0.9111
Radiation sequence with surgery (%)			0.7460
No radiation surgery	2669 (76.1)	6327 (76.3)	
Radiation before surgery	527 (15.0)	1279 (15.4)	
Radiation after surgery	277 (7.9)	624 (7.5)	
Other	33 (0.9)	67 (0.8)	
Radiation = yes (%)	1373 (39.2)	3139 (37.8)	0.1811
Chemotherapy = yes (%)	1209 (34.5)	2846 (34.3)	0.8660
Carcinomas in situ = yes (%)	430 (12.3)	1048 (12.6)	0.6043
Marita status = single/separated (%)	1466 (41.8)	3482 (42.0)	0.8944
The number of malignancies (mean (SD))	1.14 (0.42)	1.14 (0.41)	0.8211
The number of benign tumors (mean (SD))	1.00 (0.05)	1.00 (0.06)	0.6960
Median annual household income (∗10 dollars) (mean (SD))	373.03 (121.73)	371.36 (121.90)	0.4963

^a^Other (site) contains “anus,” “anal canal,” “overlapping lesion of rectum, anus, and anal canal,” and “cloacogenic zone.” ^b^Other (pathological type) contains “epithelial neoplasms,” “transitional cell papillomas and carcinomas,” and “squamous cell neoplasms”.

**Table 2:** Feature's selection.

Variables		Univariate analysis		Multivariate analysis	
		HR (95% CI)	*P*	HR (95% CI)	*P*
Number of malignant		0.93 (1.15-0.54)	1.0300		
Number of benign/borderline		0.26 (1.72-0.40)	0.6670		
Age (∗10 years)		0.892 (0.822-0.968)	0.0070^∗^		
Insurance (uninsured)		1.27 (1.16-1.40)	<0.0001^∗^	1.16 (1.06-1.28)	0.0022^∗^
Married (no)		1.42 (1.30-1.56)	<0.0001^∗^	1.23 (1.12-1.35)	<0.0001^∗^
Median annual household income (∗1000 dollars)		0.994 (0.992-0.997)	<0.0001^∗^	0.994 (0.991-0.997)	<0.0001^∗^
Race	White (ref)				
	Black	1.59 (1.41-1.80)	<0.0001^∗^	0.66 (0.58-0.74)	0.0326^∗^
	Other	1.28 (1.11-1.48)	<0.0001^∗^	0.83 (0.69-0.98)	0.0022^∗^
Sex (female)		0.93 (0.84-1.01)	0.0926		
Site	Colon (ref)				
	Rectum	0.80 (0.72-0.88)	<0.0001^∗^	0.60 (0.37-0.98)	0.0147^∗^
	Other	0.88 (0.66-1.17)	0.3640	0.54 (0.33-0.89)	0.0461^∗^
The degree of tumor differentiation	Grade I (ref)				
	Grade II	2.28 (1.79-2.91)	<0.0001^∗^	1.56 (1.22-1.99)	0.0004^∗^
	Grade III	5.27 (4.10-6.76)	<0.0001^∗^	2.80 (2.17-3.61)	<0.0001^∗^
	Grade IV	5.92 (4.34-8.07)	<0.0001^∗^	2.88 (2.10-3.95)	<0.0001^∗^
Pathological type	Adenocarcinoma (ref)				
	Cystic, mucinous, and serous neoplasms	2.00 (1.76-2.27)	<0.0001^∗^	1.57 (0.97-2.53)	0.9931
	Other	1.09 (0.83-1.43)	0.5200	1.00 (0.63-1.60)	<0.0001^∗^
Infiltration range	Localized (ref)				
	Regional	3.67 (3.07-4.40)	<0.0001^∗^	3.27 (2.72-3.92)	<0.0001^∗^
	Distant	20.40 (17.10-24.40)	<0.0001^∗^	17.58 (14.68-21.04)	<0.0001^∗^
Lymph node dissection (no)		0.97 (0.88-1.07)	0.5540		
Radiation sequence with surgery	No radiation surgery (ref)				
	Radiation before surgery	0.99 (0.87-1.13)	0.9010		
	Radiation after surgery	1.07 (0.91-1.27)	0.4120		
	Other	1.28 (0.79-2.06)	0.3190		
Radiation (no)		1.01 (0.97-1.06)	0.6150		
Chemotherapy (no)		0.97 (0.88-1.07)	0.5310		
Carcinomas in situ (no)		1.00 (0.87-1.14)	0.9740		

^∗^ *P* < 0.05.

**Table 3:** Cox proportional hazard model.

		Coef	S.E.	Wald	*P*
Race	White (ref)				
	Black	-0.4214	0.0633	-6.65	<0.0001^∗^
	Other	-0.1935	0.0898	-2.15	0.0312^∗^
Site	Colon (ref)				
	Rectum	-0.5104	0.2516	-2.03	0.0425^∗^
	Other	-0.6119	0.2504	-2.44	0.0145^∗^
Insurance (uninsured)		0.1523	0.0497	3.06	0.0022^∗^
Married (no)		0.2112	0.0476	4.43	<0.0001^∗^
Median annual household income (∗1000 dollars)		-0.0061	0.0014	-4.44	<0.0001^∗^
The degree of tumor differentiation	Grade I (ref)				
	Grade II	0.4423	0.1258	3.52	0.0004^∗^
	Grade III	1.0353	0.1294	8.00	<0.0001^∗^
	Grade IV	1.0637	0.1604	6.63	<0.0001^∗^
Pathological type	Adenocarcinoma (ref)				
	Cystic, mucinous, and serous neoplasms	0.4544	0.2435	1.87	0.0620
	Other	0.0035	0.2386	0.01	0.9884
Infiltration range	Localized (ref)				
	Regional	1.1844	0.0933	12.69	<0.0001^∗^
	Distant	2.8767	0.0917	31.35	<0.0001^∗^

^∗^ *P* < 0.05.

## Data Availability

The data used to support the findings of this study are included within the article.
